# Insights into microbe assisted remediation in plants: a brief account on mechanisms and multi-omic strategies against heavy metal toxicity

**DOI:** 10.1007/s44154-024-00168-8

**Published:** 2025-01-08

**Authors:** Arneeb Tariq, Fozia Farhat

**Affiliations:** 1 State Key Laboratory of Crop Stress Biology for Arid Areas, Shaanxi Key Laboratory of Agricultural and Environmental Microbiology, College of Life Sciences, Northwest A&F University, Yangling, 712100 Shaanxi China; 2 Department of Botany, Government College Women University, Faisalabad, 38000 Punjab Pakistan

**Keywords:** Efflux pumps, Exopolysaccharide production, Bioremediation, Biosorption, Detoxification mechanism, Plant–microbe interaction

## Abstract

Mercury (Hg), arsenic (As), cadmium (Cd), lead (Pb) and other toxic heavy metals (HM) pose significant risks to the environment, negatively impacting the morpho-physiological and biological traits of plants. At present, toxic elements constitute a significant proportion of the food chain, exerting an impact on human health due to their mobility and biomagnification. The metal exclusion biological technique stands out for its robust performance, even when dealing with extremely low metal concentrations. Its eco-friendly nature and cost-effectiveness further enhance its value. Due to the exponential growth pattern of bacteria, these exhibit high metal persistence and are recommended for metal exclusion processes. Moreover, vacuoles like vesicles present in mycorrhizal fungi can hold extremely high levels of HM. Microbe-assisted phytoremediation primarily occurs through two mechanisms: through the direct provision of the essential nutrients and phytohormones, such as plant growth regulators, siderophores, enzymes, and mineral; or indirectly by modulating the metal detoxification process. This indirect mechanism involves microbes aiding in the accumulation and sequestration of metals in plants through the secretion of specific extracellular substances like organic acids, biosurfactants, and chelators. Moreover, the metal bioavailability and translocation in the rhizosphere are also altered via various mechanisms like acidification, precipitation, complexation or redox reactions. The understanding of the molecular and physiological processes underpinning the functions of arbuscular mycorrhizal fungi (AMF) in reducing HM toxicity, improving plant performance by procuring nutrients under HM-toxicity has significantly improved in recent years. In this review, adaptive and persistent methods related to physiological and cross-protective mechanisms in bacteria and mycorrhizal fungi (MF) resulting from the evolutionary consequences of dealing with HM toxicity have been addressed. Furthermore, the article offers details on the physiological and molecular reactions of host plants with fungi, and bacteria to HM stress, which may be useful for unveiling new knowledge about the strategies of HMs remediation.

## Introduction

Heavy metal toxicity is a worldwide environmental concern that has recently gained attention (Saxena et al. 2020). Release of the hazardous wastes into soil and water, has significantly accelerated environmental pollution (Siddiqua et al. 2022). The most concerning HMs are Hg, Cr, Pb, Cd and As, since they are non-threshold poisons that have been found in higher concentrations in the aquatic, terrestrial, and aerial systems (Li et al. 2022). Due to their non-biodegradability, HMs may infiltrate into the food supply and lead to the production of contaminated foods. Heavy metals may accumulate in the cereal grains and through biomagnification might result in a range of health consequences in living organisms (Riaz et al. 2021).

Conventional treatment methods are unable to provide an economical and ecologically sound answer to address these environmental issues. Physical, chemical, and biological (bioremediation) procedures are key strategies for avoiding metal toxicity (Karaouzas et al. 2021). The physicochemical techniques are swift, but they are difficult to implement because they are costly, face technical challenges, and release toxic pollutants (Antoniadis et al. 2017). Contrarily, the less expensive and more environmentally friendly bioremediation options are the plant growth-promoting rhizobacteria (PGPR), and MF capable of HM tolerance/persistence/detoxification and producing distinct plant growth-enhancing chemicals (Mei et al. 2022).

The plant microbiome refers to the vast array of microorganisms that dwell in close proximity to plant’s root region, foliage surfaces, and other areas such as pollen and nectar (Trivedi et al. 2022). The toxicity and damaging characteristics of HM impacts microbial viability in various situations, resulting in the depletion of specific microorganisms (Rizvi et al. 2022). The substantial quantity of HM in soil–plant interface causes selection pressure for the development of susceptibility among microbiomes to harmful metals (Hao et al. 2021). Bacterial communities influence the metal complexation and transport in soil ecosystem or colonize plant surfaces, shielding plants from excess metal toxicity (Mohammadabadi and Javanbakht 2020). Enzymes released by bacteria and the entire microbial cell are thought to be eco-efficient biocatalyst for HM removal from polluted location (Aqeel et al. 2023). Endophytic fungi forms association with plants not only enhancing plant growth and adaptation but also are the key microorganisms to reduce HM toxicities in plants. Since beneficial microbial symbionts can give plants resilience to external HM pressures, they could be exploited in phytoremediation approaches in HM contaminated soil (Riaz et al. 2021).

In general, the aim of this review article is to discuss how plant root-associated microbiomes help plants to cope with the challenges confronted in HM polluted locations and to optimize plant output. Furthermore, the approaches and the advanced tools for deep insight into the mechanisms opted by the fungi and bacteria for HM tolerance have been discussed. The findings compiled from various scientists in this review article demonstrate the influence of HM (either single or dual) on numerous parameters related to plant vigor and productivity. Additionally, these findings lay the foundation for advancing combinations of metal-resistant microbes with stress-alleviating capabilities, along with a variety of plant growth enhancement abilities. This approach aims to enhance plant stability under diverse real-world environmental conditions.

## Heavy metal associated toxicity hazards to soil and environment

It is quite concerning that the environment contains a number of contaminants, such as HM, organic and inorganic suspended particles, and toxic metals (TMs). These pollutants pose detrimental impacts on general public’s health, terrestrial and aquatic biomes (Właśniewski and Hajduk 2012). The bioavailability of HM is largely dependent on the type of soil, pH, humidity, and micronutrient content. These factors all affect the dynamics of HM in the soil and how readily plants can absorb them (Gu et al. 2016). Heavy metals are persistent in soil, and when they combine with both organic and inorganic debris, they make soils more hazardous (Hu et al. 2017). As a result of this, HM can accumulate in the food web and eventually get into the body tissues via food. Moreover, HM can enter the human body through direct dietary and respiratory intake, as well as absorption through skin contact (Mohammadi et al. 2020). The primary pathways through which humans are exposed to harmful metals are inhalation of soil particles and consumption of contaminated drinking water (Alexander et al. 2006). A substantial amount of data also points to the importance of soil age in influencing plant bioavailability of metals (Smolders et al. 2009; Violante et al. 2010). The issue with HM is not only that they are harmful, but also that they can build up within living things (Zhu et al. 2011). Besides soil, HM in the atmosphere effect the crops in two ways (Xiang et al. 2021). Primarily, they directly contaminate crops by entering through the stomates of the leaves and concentrating in the cells. Additionally, HM particulates settle in the soil, build up, and inadvertently contaminate crops through their roots. Concentration of HM above threshold level in soil, atmosphere and fruits/vegetables pose adverse effects on human health upon consumption. They may lead to cancer incidences, defects in central nervous system, visual and respiratory disorders (Mahurpawar 2015; Martin and Griswold 2009).

## Heavy metals and rhizospheric microbes

Soil microorganisms, particularly those in the root zone, demonstrate a critical part for detoxifying HM contaminated soil. The removal of toxins from the soil by rhizomicrobial community is frequently known as rhizoremediation (Kuiper et al. 2004). This process entails increased metabolic performance of microorganisms particularly in plants exposed to HM poisoning. According to Pires et al. (2017), Firmicutes, Proteobacteria, and Actinobacteria make up most of the bacteria community, in HM-contaminated areas, with Bacillus, Pseudomonas, and Arthrobacter being the most prevalent species (Checcucci et al. 2017). The fungal species belongs to Ascomycetes and Basidiomycetes are the most likely organisms found in HM polluted terrestrial ecosystem (Narendrula-Kotha and Nkongolo 2017). Metal microbe interactions in the rhizosphere are highly strict and are influenced by the soil’s physical and chemical characteristics, the number of metallic elements, metabolous procedures, and the microbial communities (Mei et al. 2022).

### Mechanisms of HM Remediation by HM Tolerant Plant Growth Promoting Microbes (HMT-PGPM)

The availability and transport of HM from the soil into roots are crucial factors in mitigating their harmful effects on plants (Zhong et al. 2020). Heavy metals cannot be degraded but only modified from one chemical state to another, which alters their mobility and potency (Santona et al. 2006). Metals are either manipulated through redox process or via alkylation. Microorganisms can also acquire metals through mechanisms that are either metabolism-dependent or metabolism-independent. (Al-Dhabaan 2019). At the moment, the HM-tolerant microorganisms primarily include fungal, bacterial species, and their consortium. These factors may alters metal translocation indirectly by changing pH, primarily by producing specific chemicals which change the uptake ability of the metals by the plants (Wani et al. 2023).

**Fig. 1:**
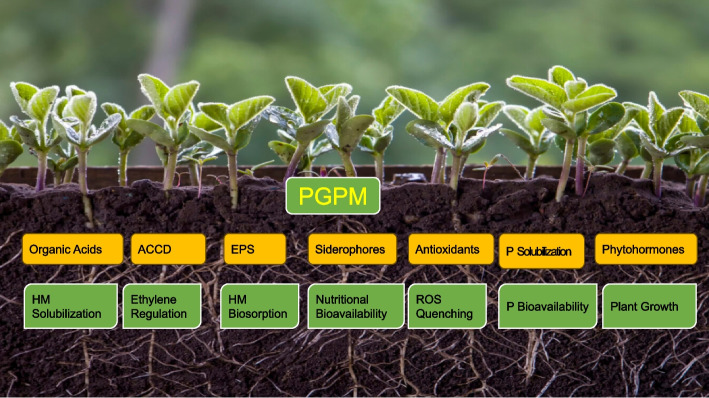
Functions of PGPM to support plants against HM toxicity. ACCD 1-aminocyclopropane-1-carboxylate deaminase, EPS Extracellular polymeric Substances, ROS Reactive Oxygen Species, P Phosphorous

The primary mechanism of their action involves 1) HM-microbe linkage, therefore arresting the HM restricting their transport in the plant roots 2) production of growth-boosting molecules (growth regulators, organic acids and ACC deaminase), 3) making the soil chemical and physical profile feasible, 4) the microbial consortia, 5) altering the movement procedure of toxic metals from soil into plants, and 5) antioxidant improvement (Wang et al. 2022) (Fig. 1). Certain microbial species involved in HM tolerance of plants and their mechanism of action are ascribed in Table 1.

**Table 1:** Microbes used in HM alleviation through different mechanisms

Microbe	Heavy Metal Stress Alleviation	Source of extraction	Mechanism	Reference
*Serratia marcescens* *Rhodotorula mucilaginosa*	Zn, Cd, Pb	Contaminated soil	IAA, ACC Deaminase Siderophores	(Ji et al. 2012)
*Polygonum pubescens* *Rahnella* sp	Zn, Cd, Pb	*Brassica napus*	IAA, ACC Deaminase Siderophores	(He et al. 2013)
*Enterobacter* sp. *Klebsiella* sp.	Zn, Cd, Pb	Contaminated soil	IAA	(Jing et al. 2014)
*Pseudomonas putida* *B. safensis* *Phylllnathus urinaria*	HM	Contaminated soil	IAA	(Ratan et al. 2015)
*Pseudomonas protegens*	HM	Contaminated soil	IAA, chitinase, polymer degrading enzymes, and siderophores	(Bensidhoum et al. 2016)
*Staphylococcus arlettae*	HM	*Helianthus annus L*	Phytohormones	(Qadir et al. 2020)
*Leifsonia xyli*	Cu	*Solanum lycopersicum*	Gibberellins and IAA	(Kang et al. 2017)
*Paecilomyces formosus* and *Sphingomonas* sp (Cosortium)	Al, Zn	*Glycine max*	Gibberellins	(Bilal et al. 2018)
*Penicillium roqueforti*	HM	*Triticum aestivum L*	IAA	(Ikram et al. 2018)
*Penicillium funiculosum*	HM	*G. max*	IAA	(Bilal et al. 2019)
*P. formosus*	HM	*G. max*	IAA Gibberellins	(Bilal et al. 2021)
*P. funiculosum*	HM	*G. max*	IAA Gibberellins	(Bilal et al. 2021)
*Pantoea agglomerans* *Pseudomonas thivervalensis*, *Ralstonia* sp.	Cu	*B. napus*	ACC Deaminase, Siderophores	(Zhang et al. 2011a)
*Penicillium citrinum, Phytophthora sojae* and *Trichoderma asperellum*		*Contaminated Soil*	ACC Deaminase	(Deng et al. 2012)
*Burkholderia* sp	Cd, Zn	*Salix caprea*	Siderophores, IAA, ACCD	(Kuffner et al. 2010)
*Agrobacterium tumefaciens* and *Bacillus* sp	Pb	*Commelina communis*	Siderophores, IAA, ACCD	(Zhang et al. 2011b)
*Pseudomonas* sp	Pb, Cd	*Oudemansiella radicata*	Siderophores	(Cao et al. 2012)
*Bacillus amyloliquefaciens*	As, Pb, Al, Cd	*HM Contaminated soil*	Siderophores	(Gaonkar and Bhosle 2013)
*Acinetobacter* sp. and *P. putida*	Cu	*Zea mays* L	Siderophores, IAA, and phosphorus solubilization	(Rojas-Tapias et al. 2014)
*Streptomyces lividans* and *Streptomyces mirabilis*	HM	*Plants*	Siderophores	(Schütze et al. 2015)
*Brevundimonas diminuta*	As	*Oryza sativa* L	Siderophores	(Devi et al. 2022)
*Enterobacter* sp	Fe	*Hibiscus cannabinus*	Siderophores IAA	(Chen et al. 2017)
*Klebsiella pneumonia*	Cd	*Vigna munga*	Siderophores	(Dutta et al. 2018)
*Pseudomonas orientalis* and *Chaetomium cupreum*	HM’s	*Eucalyptus globulus*	Siderophores	(Ortiz et al. 2019)
*Pseudomonas* and *Bacillus*	Cd, Pb, Zn	*Spinacia oleracea*	Siderophores	(Shilev et al. 2020)
*Consortium of Rhizobium* fungi *Piriformospora indica*	Cd	*Medicago sativa*	Siderophores	(Sepehri and Khatabi 2021)
*P. aeruginosa*	Cd	*T. aestivum* L	Increased soluble protein, chlorophyll content, plant fresh and dry weight and root elemental acquisition	(Islam et al. 2014)
*Eichhornia crassipes*	Cd, Zn	*Plants*	Phosphorus solubilization, Siderophores and IAA production	(Priya et al. 2022)
*Trichoderma virens*	Fe	*Contaminated soil*	Phosphorus solubilization and exhibiting the phytase activity Siderophores ACCD	(Devi et al. 2022)
*Paenibacillus yonginensis*	Al	*Arabidopsis thaliana*	Phosphate solubilizer, and siderophores producer	(Sukweenadhi et al. 2015)
*B. thuringiensis*, *B. cereus*, *B. subtilis* and *Stenotrophomonas maltophilia*	Cr	*Cicer arietinum*	Phosphorus and potassium solubilizing	(Shreya et al. 2020)
*B. subtilis*, *P. aeruginosa*, and *Torulopsis bombicola*	HM alleviation	*HM Contaminated Soil*	Di-rhamnolipids, rhamnolipids, and sophorolipids	(Juwarkar et al. 2007; Venkatesh and Vedaraman 2012)
*Bacillus Sp*	hexavalent Cr	*HM Contaminated Soil*	Synthesis of biosurfactants and extracellular enzymes	(Gnanamani et al. 2010)
*Escherichia fergusonii*	Remediated Cu, Mn, Pb, Fe, Ni, and Zn	*HM Contaminated Soil*	Lipopeptide Biosurfactants	(Sriram et al. 2011)
*Candida lipolytica*	Remediated Cu Zn Cd Fe	*Oil contaminated soil*	Lipoprotein Biosurfactant	(Rufino et al. 2012)
*B. subtilis*	Cd, Pb, Ni, Cu, Co, and Zn biodegradation	*Waste water*	Biosurfactant consisting fengycin and surfactin	(Singh and Cameotra 2013)
*Citrobacter freundii*	Bioremoval of HM	*Waste waster*	Biosurfactant	(Gomaa and El-Meihy 2019)
*P. putida*	arresting Cd^2+^ into organic complexes	HM Contaminated Soil	EPS	(Xu et al. 2012a)
*Azotobacter* spp	inhibit Cd and Cr absorption	HM Contaminated Soil	EPS	(Wei et al. 2011)
*Desulfovibrio desulfuricans*	Cu^2+^, Cu^2+^ and Zn^2+^	*T. aestivum* L	EPS	(Joshi and Juwarkar 2009)

### Solubilization of HM by organic acids

Fumaric, malic, oxalic, and citric acids crucial cellular ligands for Zn, Cd, and Ni and associated with HM tolerance in numerous plant species. Bacteria and AMF produce organic acids that aid in solubilization by providing protons and metal complexing anions in organic acids. This leads to the release of hazardous metals (loids) from contaminated soil through ion exchange and a protons-facilitated mechanism, a phenomena known as bioleaching (Sarkodie et al. 2022). The oxidation, solubilization, and complexation of hazardous metal(loid)s in soil facilitated by the metabolic reactions and the production of primary products are the main foundations of the bioleaching process (Johnson 2018). The presence of HMT-PGPM and the secretion of organic acids also balances the soil pH and separates soluble metallic ions from other compounds (Dighton and White 2005). Guo et al. (2007) demonstrated that excessive Cd absorption leads to the increased secretion of organic acids including malate and succinate, and they operate as a possible Cd ligand, limiting further damage. Experimental evidence suggests that a wide array of microorganisms secrete numerous organic compounds as innate HM binding agents (Seneviratne et al. 2017). Gluconic, malic, acetic and oxalic acids are the major compounds produced by soil microorganisms for HM detoxification (Gube 2016). Mycorrhizal fungi especially *Pencillium* and *Aspergillus* oxalate crystals have also been shown to lock and minimize the effects of HM (Mishra et al. 2017). Their mycelial filament chelates or absorbs HM in deeper soil aggregates. An experiment conducted by Kaewdoung et al. (2016) reported that oxalate formed by wood-rotting fungi *Fomitopsis cf. meliae* and *Ganoderma aff. steyaertanum* is involved in inducing HM resistance by lessening the toxicity of the HM (sulfate compounds of Zn, Cd and Cu into their hydrated oxalates).

### Synthesizing ACCD (1-aminocyclopropane-1-carboxylate deaminase) to reduce ethylene production

Ethylene biosynthesis enhanced in HM exposed plants, causing root elongation declination, apoptosis, and hydrogen peroxide accumulation (Zhong et al. 2020). The ACCD production by microorganisms is widely acknowledged way for inducing HM tolerance in plants. The enzyme ACCD catalyzes the hydrolysis of 1-aminocyclopropane-1-carboxylate (ACC), which is an immediate precursor of ethylene formed under high-stress conditions. This hydrolysis process yields ammonia and α-ketobutyrate, thereby enhancing root growth in plants and reducing the concentration of ethylene within the plant. To efficiently reduce HM stress in plants, a diverse group of microorganisms have been identified involved in synthesizing ACCD (Singh et al. 2021). Thus, bacterial strains which have ACCD activity can partially prevent any reduction in the length of plant roots and shoots, biomass and any other physio-biochemical damage caused by high ethylene levels particularly those resulting from HM stress (Grichko and Glick 2001; Mayak et al. 2004; Penrose and Glick 2003). The ACCD producing bacteria, present in the rhizosphere were found to be very substantial in improving the characteristics of plants growing under stress, including plant development, retention of water, cell membrane equilibrium, synthesis of compatible substances and protein, phenolic levels, chlorophyll and carotenoids (Tiwari et al. 2018).

### *Biosorption of HM *via* secretion of extracellular polymeric substances*

Extracellular Polymeric Substances are natural polymers with high molecular weight that are released into the environment by bacteria. They are composed of homo and heteropolysaccharides (mucopolysaccharides, humic proteins, and substances) that cling to the microbial cell membranes (Wingender et al. 1999). The polysaccharide content of EPS differs between microbe species; however, the most expressed EPS by microbes against HM toxicity are derived amino sugars, natural sugars, carboxylic ester group, pyruvate ketals, and uronic acid (Yadav 2021). Exopolymers' ionic nature is ascribed to their alkane carbonyl carbon atom, which decreases the compound's lipophobicity and, as a result, modifies the reaction between specific compound and other cations (Kaushal and Wani 2016). Microbial EPS are crucial in forming complexes with deleterious HM and lowering their translocation within plant cells (Rajkumar et al. 2012). It has been shown that the EPS of PGP rhizobacteria binds potentially dangerous trace elements and precipitate the metal oxides and sulphides, promoting the formation of organic metal complexes and boosting HM resistance (Xu et al. 2012a). Certain PGP bacteria produce EPS in response to harmful HM exposure, which triggers the formation of biofilm. Through the creation of a protective layer and the transformation of hazardous metal ions into non-toxic forms following adsorption, biofilm growth increases microbial cell tolerance. It has been demonstrated that many HM are sequestered through anionic group containing EPS produced by rhizobia and other PGP bacteria (Gupta and Diwan 2017).

### Production of siderophores

Both microorganisms and plants manufacture small secondary metabolites called siderophores, which are iron-chelating molecules that are used to sequester iron from the surrounding rhizosphere (Ghosh et al. 2020a). This chemical aids in the fight against nutritional deficiency (particularly iron), abiotic (HM stress) and biotic (pests and pathogens) stressors. There are four categories for these non-ribosomal peptides: catecholate, hydroxamate, carboxylate, and mixed forms (Khan et al. 2018). Even though several siderophores have been reported, others still need to be characterized chemically and functionally (Ghosh et al. 2020b). The siderophores' most well-known function is to mobilize iron and make it accessible (Schwabe et al. 2020), converting insoluble Fe^3+^ to soluble form Fe^2+^. A particular receptor protein on the surface of microorganisms recognizes the ferric-siderophore complex, which is then actively transported through plasma membrane where it is reduced to a soluble ferrous form (Khan et al. 2018; Nosrati et al. 2018). When it comes to this process, certain siderophores have an affinity for divalent and trivalent cations, like Pb^2+^, Zn^2+^, Ni^2+^, and Al^3+^. Consequently the HM-siderophore compounds are produced which are comparatively less harmful (Albelda-Berenguer et al. 2019). While some HM can hinder plants' ability to absorb iron from the soil, siderophores have been shown to be able to do so when Al, Cu, Mn, Ni, and other metals are present (El-Maraghy et al. 2020). Apart from their function in the intake of metals, siderophores may also play a role in the uptake of iron decreasing the accumulation of reactive oxygen species (ROS) within the cell (Rajkumar et al. 2010).

As a result, microbes that produce siderophores can boost plant development, improving iron feeding when plants are starving and shielding plants from HM-induced oxidative damage (Seneviratne and Vithanage 2015). So, it makes these compounds an essential in the plant survival in environments contaminated with HM, which should be used in phytoremediation with the help of microbes (Kumar et al. 2021; Rai et al. 2020).

### Induction of antioxidant enzymes by the plants

Exposure to HM stress prompts plants to release ROS and cyto-lethal substances such as methylglyoxal (MG), disrupting the ionic homeostasis within plant cells (Sytar et al. 2013). In response, the plant releases a range of non-enzymatic (alpha-tocopherol and ascorbate) and enzymatic (ascorbate peroxidase, catalase, dehydroascorbate reductase, glutathione reductase, glutathione-S-transferase, glutathione peroxidase, guaiacol peroxidase, and superoxide dismutase) antioxidants to guard against oxidative stress (Miller et al. 2010). Plant growth promoting rhizobacteria may reside superficially on the plant roots and ameliorate the HM adversities by supplying minerals like nitrogen (N), phosphate (P), and potassium (K). They also elevate the stress by synthesizing the plant growth promoting hormones like auxin (IAA), cytokinin (CK), and abscisic acid (ABA) (Neshat et al. 2022). A study evaluated the effectiveness of PGPR strains *Acinetobacter beijerinckii* and *Raoultella planticola* in mitigating the impact of Cr and As stress on soybean seedlings (Husna et al. 2023). Compounds synthesized nonenzymatically, like proline (Pro), can enhance the metal-detoxification ability of intracellular antioxidant enzymes (Emamverdian et al. 2015). Metal exposure may result in the reduced synthesis of some essential stress regulators by rhizobacteria. This suggests that the strains may accumulate within their cells to efficiently scavenge ROS (Hayat et al. 2012). According to several studies, PGP microorganisms stimulate plants to generate these antioxidants and other metabolites in response to metal toxicity (Table 1).

### Phosphorus solubilization by HMT-microbes

Another microbial assisted mechanism to shield plants against HM toxicity is phosphorus (P) solubility. Microorganisms have the potential to dissociate P-complexes such as Fe-P, Al-P and Ca-P naturally present in rhizosphere, enhancing phosphorus availability and quickly immobilizing HM in soil (Pinter et al. 2017). The HM immobilization via insoluble HM-P complex production, plant tolerance to HM stress can be enhanced. Many microbial genera have been discovered in the literature to demonstrate this mechanism and alleviate HM stress (Yadav et al. 2015). Various microbial extracts were found to have P-solubilization, siderophores, ACCD, and IAA production that effectively reduced Cd stress and enhanced *S. nigrum* growth in a study conducted by Chen et al. (2010). Many other plant species also showed HM toxicity persistence by modulating growth and physiological attributes induced by microbes (Rawat and Tewari 2011).

### Mediation of Bio-surfactants by HMTM (HM tolerant microbes)

Biosurfactants are diverse range of surface-active amphipathic chemicals mainly comprised of both hydrophobic and hydrophilic components such as phospholipid, fatty acids, lipoprotein, mycolic acid and glycolipids (Pacheco et al. 2010). Several types of bacteria create them as metabolites at different stages of their growth. Biosurfactants in microbes have been discovered to have several advantages in terms of pollutant biodegradability, especially HM (Pacwa-Płociniczak et al. 2011). Hazardous HM cleanup mediated by biosurfactants is encouraged in solid phases. By strengthening their bond with positively charged metal ions during the ion exchange process, negatively charged biosurfactants make the metal ions non-toxic and reduce metal toxicity in the rhizosphere (Chakraborty and Das 2014).

### Efflux-mediated heavy metal resistance

Efflux pumps are very crucial in eliminating HM and maintaining essential homeostasis. Currently, there is a widespread acknowledgment of the role of environmental factors in fostering acquired resistance among pathogenic bacteria, which occurs in four distinct phases: the emergence of new resistance genes, their mobilization, transfer to pathogens, and subsequent dissemination. Conversely, acquired resistance mechanisms typically arise through horizontal gene transfer (HGT) and encompass plasmid-encoded specific efflux pumps, such as TetK and TetL found in *S. aureus* (Peterson and Kaur 2018). These metals often infiltrate cells through the same transport channels used by essential elements. For instance, in *Ralstonia metallidurans*, chromium exploits the sulfate transport system, facilitating the entry of Cd, Zn, Co, and Ni ions. Similarly, manganese utilizes the magnesium transport system (Nies et al. 1989). Arsenic-resistant microbes like *Escherichia coli *employ GlpF and phosphate transporters (Pst and Pit pumps) for the uptake of arsenate and arsenite (Rosen 2002). The excessive buildup of heavy ions is mitigated by energy-dependent efflux systems. In *Escherichia coli*, copper efflux is regulated by the RND CusCBA multiprotein complex. Similarly, *Bacillus sp*. SFC 500-1E employs a chromate ion transporter protein called ChrA to expel toxic chromate ions (Cr (VI)) (Franke et al. 2003). Research has identified efflux determinants of resistance, which aid in recognizing potential resistance mechanisms through cloned genes and in vitro strain construction (Poole 2007; Sun et al 2014).

## The cellular level multi-omics response of plant-HMTM interaction

**Fig. 2:**
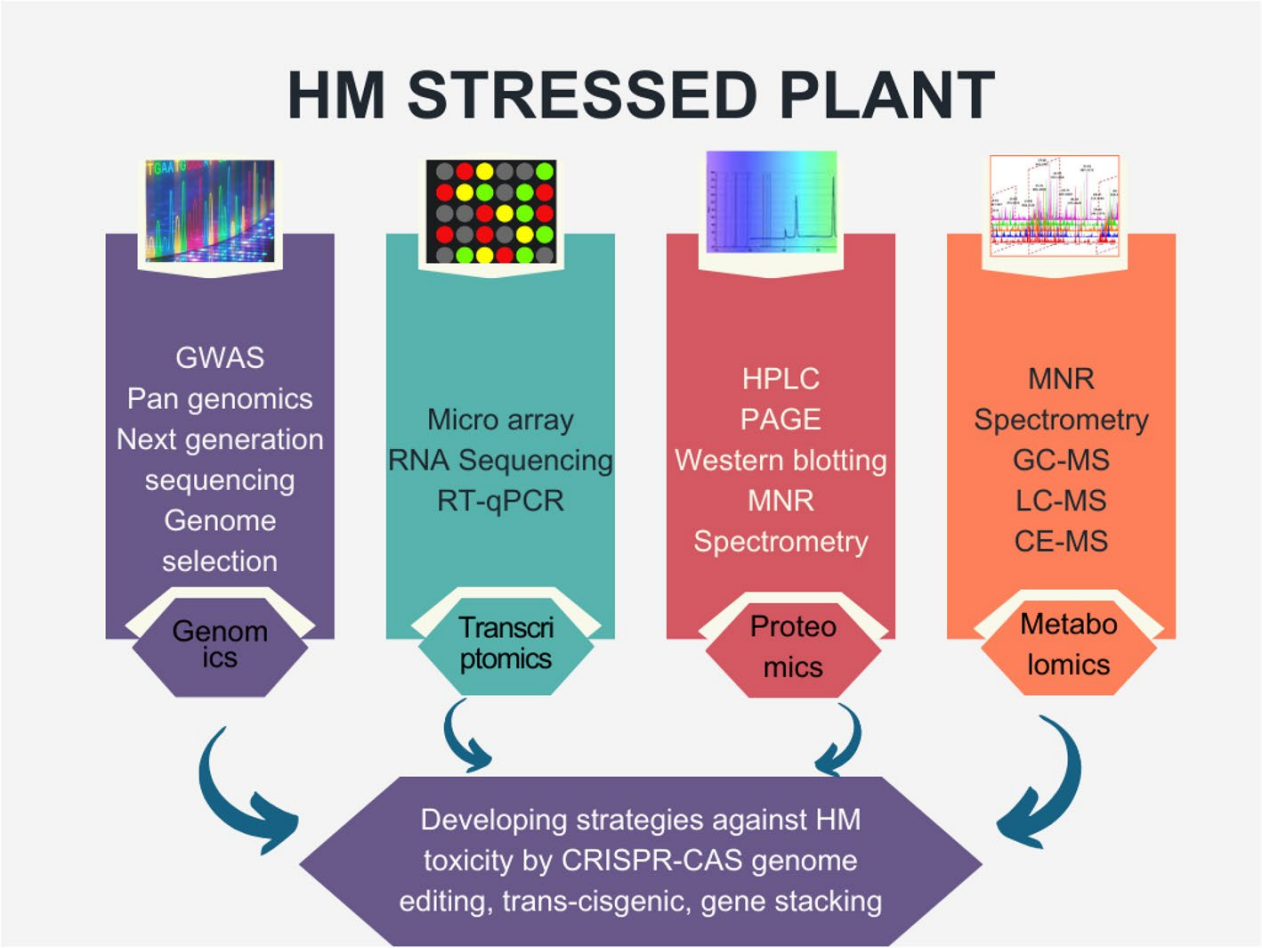
Omic approaches in mitigating HM toxicity in plants. RT-PCR Reverse transcription polymerase chain reaction, HPLC High Performance Liquid Chromatography, PAGE Poly Acrylamide Gel Electrophoresis, MS Mass Spectrophotometry, NMR Nuclear Magnetic Resonance, GC–MS Gas Chromatography- Mass Spectrometry, LC–MS Liquid Chromatography- Mass Spectrometry, CE-MS Capillary electrophoresis–mass spectrometry

Plant–microbe interactions are intricate and complex, but they are highly calibrated. To get the most out of this relationship, comprehension signals ligands generated by both partners. Plant–microbe interface demonstrates numerous physio-biochemical reactions at cellular as well as molecular level. Understanding the transformations occurring in plant genome, proteome, transcriptome, or metabolome is a challenging task, which can be facilitated by employing multi-omics approaches (Fig. 2). Recently developed meta-omic approaches offer effective means of exploring microbial populations and their roles in stressful environments (De Castro et al. 2013).

### Metagenomics

Understanding the genetic regions influencing HM-related characteristics, a deeper investigation of plant-microbial interface, tolerance and the availability of molecular markers are required which has been briefed in the Fig. 2. Metagenomic approaches are sources to provide habitat-specific dispersal of microbes with production of antibiotics and plant growth stimulating substances (Xu et al. 2012b).

In-depth understanding of the relationship between Trichoderma species (*T. atroviride* and *T. harzianum*) and *Lycopersicum esculentum* highlights the significance of plant genetic traits in alleviating stress. This is achieved through the modulation of IAA metabolism and the ACC deaminase pathway within the root zone (Gravel et al. 2007). RNA interference was used to validate an ACC-deaminase sequence that was thought to be accessible in the *Trichoderma* genome (Kubicek et al. 2011). The polymerase chain reaction (PCR) analysis revealed expression of two ACC-deaminase genes (acdS) similar to that of *Pseudomonas fluorescens* for alleviating HM imposed adversities in a study on potato endophytes. Analysis of clones in metagenomic libraries aided in the identification of the full acdS operon from an uncultivated endophyte and indicated the presence of acdR (a regulator gene) towards 5' end relative to acdS (Nikolic et al. 2011).

Metagenomics provides insights into the effectiveness of microbial genomes by revealing the number of genes involved in stress-alleviating metabolic pathways. Through diversity profiling and colonization studies using metagenomics, it becomes possible to quantify the extent of colonization by a specific host in the presence of stressors (Turner et al. 2013).

### Transcriptomics

Identifying discrete transcript groups that contribute to the variation between two biologically distinct expressions under various environmental conditions (stressed and non-stressed) can be done by comparing transcriptome profiles (Bräutigam and Gowik 2010). Two crucial techniques for evaluating plant–microbe interactions are the transcriptome-level information generated by mRNA sequencing analysis and the microarray technology. Next-generation RNA sequencing revealed that the stress-responsive genes were elevated in strains of *Sinorhizobium meliloti* that overproduced IAA (Defez et al. 2016). In a comparison of transcription profiles between the wild-type strain of *S. meliloti* (strain-1021) and an IAA overproducing derivative (RD64 strain), the latter exhibited upregulation of stress-responsive genes and genes encoding sigma factors. Additionally, the transcriptomic profile of the symbiotic association between rapeseed and *Stenotrophomonas rhizophila* identified spermidine as a newly discovered plant growth hormone (Alavi et al. 2013). MicroRNAs (miRNA) besides TFs also regulate stress signaling mechanisms, which plays vital role in root and leaf morphogenesis (Curaba et al. 2014). In *B. vulgaris*, 13 miRNAs were identified by using an in-silico approach (Li et al. 2015). MiR398 is involved in regulating the expression of superoxide dismutase mRNAs, specifically SOD1 and SOD2, contributing to the reduction of ROS and mitigating the subsequent effects of environmental stress (Kantar et al. 2011). To reduce stress, several forms of miRNAs control a variety of cellular reactions and metabolic activities, such as apoptosis, ion transport, auxin balance, and transcriptional regulation (Li et al. 2010). Aluminium stress tolerance mechanism exhibited differently by two different rice subspecies, *japonica* and *indica*, miRNA has also been noticed to modulate the plant response to aluminium stress. The use of RT-qPCR identified 16 distinct miRNA responses, demonstrating a complicated response to aluminium stress (Lima et al. 2011).

### Proteomics

Proteins are important in expression of stress response in plants because they directly represent the phenotypic characteristics. As a result, proteomic investigations have evolved into formidable tools for investigating metabolic pathways and protein–protein communications in microorganisms and plant cells (Silva‐Sanchez et al. 2015). Plants in microbial association can aid in pinpointing the protein targets and connections with stress and without stress. The use of 2-DE- proteomics tool revealed the *Pseudomonas sp*. TLC 6–6.5–4 activity in increasing the maize fresh/dry weight and improved resistance to metal stress. By using 4-DE, a more advanced approach further revealed 498 high-abundance protein sites involved in different repair and biosynthetic pathways (Li et al. 2014).

The host's tolerance to potentially hazardous levels of soil, zinc, and other toxic metals has been improved by AM fungus. Comparative proteomic study has also been used to examine AMF-mediated Cd stress tolerance in *M. truncatula*. Mycorrhizae symbiosis enhanced photosynthesis-related protein production while decreasing glycolysis and altering antioxidation pathways in the plants (Li et al. 2023). Proteomic data of rhizospheric microbiota of *Biscutella laevigata* and *Noccaea caerulescens* in naturally HM contaminated serpentine soil identifies several proteins responsible for nutrient uptake and metal compartmentalization to avoid toxicity in the cell (Mattarozzi et al. 2017).

### Metabolomics

The study of every metabolic process and product that a cell produces is known as metabolomics. Significant variety is seen in the cellular metabolism in response to environmental changes around an organism (Wishart 2019). It is consequently critical to have extensive information of an organism's metabolomics in both stressed and without stressed states, by which the induction/suppression of typical hallmark compounds of interest can be inferred (Anzano et al. 2021). This will help detect modifications made to the pathways and the overexpression of particular stress-responsive genes (Bundy et al. 2005).

For example, a targeted metabolomics method was used to study the Pb resistance capacity of *Salix integra* after infected with various rhizobacteria. Contamination with Pb-resistant *Bacillus* sp. and *Aspergillus niger* enhanced proline content and enzymatic antioxidant. In addition, 410 metabolites, primarily organic acids, amino acids, and carbohydrates, were discovered after microbial contamination in metal toxicity (Niu et al. 2021). Another recent study looked into the mechanism of improved wheat tolerance to HM stress after being infected with the *Enterobacter bugandensis* (a toxic metal immobilizing bacterium). This microbe mitigated HM stress by a variety of methods, including 50% less HM uptake, improved bioprecipitation, extracellular absorption, and secretion (Han et al. 2021).

The combined inoculation of *Agrobacterium tumefaciens* CCNWGS0286 (an auxin synthesizing strain) and *Sinorhizobium meliloti* (N-fixing strain) in *Medicago sativa* raised the essential nutrients uptake efficiency, in addition to fostering plant growth by promoting the root nodulation, plant biomass and antioxidant activity (Jian et al. 2019). A similar study on the *Bacillus subtilis*-mediated Cd stress alleviation was reported in *M. sativa*. The growth and Cd accumulation efficiency was enhanced upon contamination through rise up in antioxidant enzyme activity and reduced cell membrane damage in plants (Li et al. 2019).

## Conclusion and future prospects

The great success of phytoremediation primarily depends on the plants' potential for ion uptake, translocation, and more biomass production in HM contaminated soils as well as the bioavailability of contaminants. Previous research findings have recommended exogenic usage of phytohormones producing microbes as an inventive strategy to enhance the HM tolerance in plants. Besides the release of phytohormones, these microorganisms can also expand plant development and productivity by either direct or indirect mechanisms. In a nutshell, mycorrhizal fungi and bacterial assisted remediation is a sustainable method and have the beneficial role to regulate the endogenous hormonal synthesis of plants. Numerous scientific works have been performed to address sustainable agriculture production. However, for efficient and long-lasting remediation of HM, additional study must be conducted.

1. Further investigations are required to classify the expedient soil microorganisms competent to generate HM stress resistance/tolerance with varied physico-chemical soil qualities under various environmental situations.
2. Omics, computational and bioinformatic approaches should also be used to inspect the suitable operational time to determine the host plants' receptors for the expression of genes after inducing microbial inoculum, interactions between plants and microbes in order to increase yields and soil and ecosystem wellness.
3. Using genetic modification techniques, such as CRISPR/Cas9, it is possible to increase the natural ability of plant-associated endophytes to tolerate and mitigate HM.
4. Apart from all these efforts, additional adaptive and cross-field trials are recommended for authenticating the sustainable bioremediation technique to alleviate the HM contamination.
5. For effective use in agricultural productivity and ecological survival, knowledge deficits in the genetic architecture of bacteria have yet to be explored and filled.

## Data Availability

All the data is included in the manuscript.

## Abbreviations

HM: Heavy metal
Cd: Cadmium
As: Arsenic
Hg: Mercury
Cr: Chromium
Cu: Copper
Al: Aluminum
Fe: Ferric
Mn: Manganese
Co: Cobalt
Pb: Lead
Zn: Zinc
Ni: Nickle
P: Phosphorus
IAA: Indole acetic acid
AMF: Arbuscular mycorrhizal fungi
MF: Mycorrhizal Fungi
PGPR: Plant growth-promoting rhizobacteria
HMT-PGPM: Heavy metal tolerance plant growth promoting microbes
ACCD: 1-Aminocyclopropane-1-carboxylate deaminase
EPS: Extracellular polymeric Substances
mRNA: Messenger ribonucleic acid
miRNA: Micro ribonucleic acid
SOD: Superoxide dismutase
TFs: Transcription factors
RT-qPCR: Real time quantitative polymerase chain reaction
DE: Dimensional electrophoresis
CRISPR/Cas9: Clustered regularly interspaced short palindromic repeats/ CRISPR-associated protein 9
